# Neuroprotective effect of hydrogen sulfide against glutamate-induced oxidative stress is mediated via the p53/glutaminase 2 pathway after traumatic brain injury

**DOI:** 10.18632/aging.202575

**Published:** 2021-02-26

**Authors:** Jianping Sun, Xiaoyu Li, Xiaoyu Gu, Hailong Du, Gengshen Zhang, Jianliang Wu, Feng Wang

**Affiliations:** 1 Department of Neurosurgery, The Second Hospital of Hebei Medical University, Shijiazhuang 050000, Hebei, P.R. China; 2 Department of Thyroid and Breast Surgery, The Second Hospital of Hebei Medical University, Shijiazhuang 050000, Hebei, P.R. China

**Keywords:** traumatic brain injury, hydrogen sulfide, glutamate, oxidative stress, p53

## Abstract

Several reports suggest that hydrogen sulfide (H_2_S) exerts multiple biological and physiological effects on the pathogenesis of traumatic brain injury (TBI). However, the exact molecular mechanism involved in this effect is not yet fully known. In this study, we found that H_2_S alleviated TBI-induced motor and spatial memory deficits, brain pathology, and brain edema. Moreover, sodium hydrosulfide (NaHS), an H_2_S donor, treatment markedly increased the expression of Bcl-2, while inhibited the expression of Bax and Cleaved caspase-3 in TBI-challenged rats. Tunnel staining also demonstrated these results. Treatment with NaHS significantly reduced the glutamate and glutaminase 2 (GLS-2) protein levels, and glutamate-mediated oxidative stress in TBI-challenged rats. Furthermore, we demonstrated that H_2_S treatment inhibited glutamate-mediated oxidative stress through the p53/GLS-2 pathway. Therefore, our results suggested that H_2_S protects brain injury induced by TBI through modulation of the glutamate-mediated oxidative stress in the p53/GLS-2 pathway-dependent manner.

## INTRODUCTION

Traumatic brain injury (TBI), which contributes to subsequent damage of associated neurons, has imposed a significant burden on family and society in the world [1, 2]. Although great improvements have been made in medical intervention, many approaches or neuroprotective agents for TBI failed during clinical trials [3–5].

Glutamate has been reported to play an important role in the excitement of nerve activity during TBI [6, 7]. Garzón et al showed that NeuroEPO protected cortical neurons from glutamate-induced apoptosis via the upregulation of Bcl-2 and inhibit glutamate-induced activation of caspase-3 [8]. Glutathione depletion, perhaps triggered by early glutamate-mediated excitotoxicity, led to late post-repetitive mild TBI loss of parvalbumin-positive interneuron-dependent cortical inhibitory tone [9]. Also, zolpidem prevented glutamate-induced toxicity in differentiated P19 neurons via the PI3K/Akt pathway [10]. Therefore, searching for neuroprotective agents, which can inhibit glutamate-induced toxicity, may be a promising therapeutic strategy for TBI treatment.

As an important gasotransmitter and endogenous neuromodulator, hydrogen sulfide (H_2_S) has been reported to exert multiple biological and physiological effects on the pathogenesis of numerous diseases, such as stroke, Alzheimer’s disease, and TBI [11–13]. Sodium hydrosulfide (NaHS), an H_2_S donor, improved spatial memory impairment of rats with TBI [14]. H_2_S could prevent scratch-induced cellular injury, alteration of mitochondrial membrane potential, intracellular accumulation of reactive oxygen species, and cell death in PC12 cells through modulation of the PI3K/Akt/Nrf2 pathway [15]. Besides, NaHS treatment increased endogenous antioxidant enzymatic activities and decreased oxidative product levels in the brain tissue of TBI-challenged rats [16]. However, the biological role of H_2_S in regulating glutamate-induced apoptosis and oxidative stress during TBI remains unclear. In the present study, we used the TBI rat model to determine the protective effects of H_2_S on the motor and spatial memory deficits and brain edema. Furthermore, we explored its potential neuroprotective mechanism through glutamate-induced apoptosis and oxidative stress.

## RESULTS

### H_2_S alleviated TBI-induced motor and spatial memory deficits and brain edema

**Figure 1:**
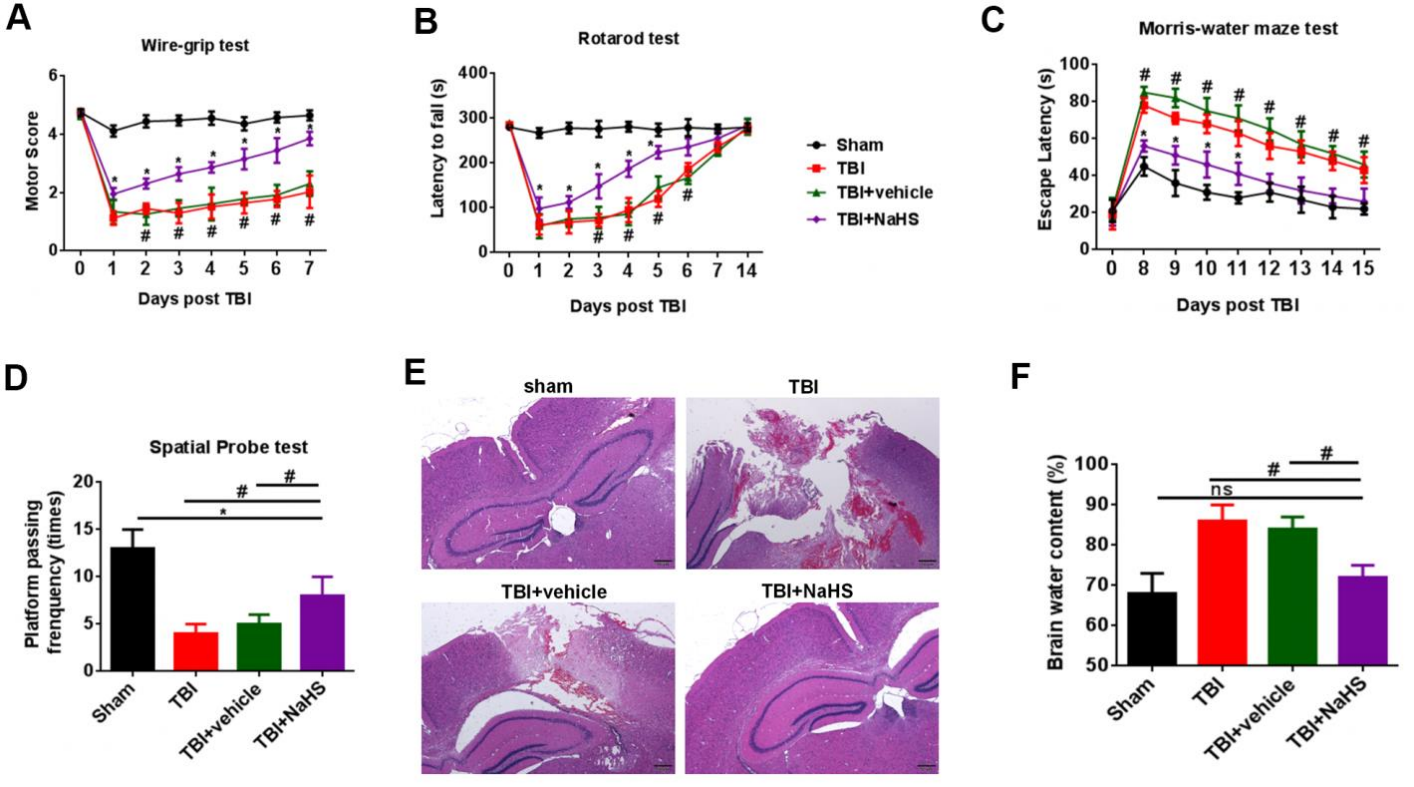
**H**_2_**S alleviated TBI-induced motor and spatial memory deficits and brain edema.** (**A**) A wire-grip test was performed to analyze the motor function at 0 to 7d after TBI (n = 5). (**B**) A Rota-rod test was performed to analyze the motor function at 0 to 7d and 14d after TBI (n = 5). (**C**) A Morris water maze test was performed to test spatial memory ability on days 8 to 15 (n = 5). (**D**) A spatial probe test was performed to test spatial memory ability on days 16 (n = 5). (**E**) The pathological changes was analyzed by H&E staining. (**F**) The brain water content was measured at 24 h after TBI (n = 5). #P<0.05 vs. sham group.* P<0.05, vs. TBI and TBI+ vehicle groups. #P<0.05 vs. sham group.

To determine the effect of H_2_S on TBI-induced motor deficits, a wire-grip test and a rota-rod test were performed. Compared with the sham group, TBI lead to a significant decline in motor performance at 0 to 7 days post-injury. NaHS, an H_2_S donor, treatment significantly improve the motor function on days 0 to 7 after TBI compared to TBI and TBI+ vehicle groups (Figure 1A, 1B).

We then performed a Morris water maze test to analyze spatial memory ability on days 8 to 15. Compared with the sham group, rats from TBI and TBI+ vehicle groups showed increased latencies to find the hidden platform. We observed a significant decrease in the latencies in the TBI+ NaHS group (Figure 1C). The data of a spatial probe test also showed that H_2_S improved the spatial memory ability of TBI rats (Figure 1D).

To determine the effect of H_2_S on TBI-induced cerebral injury, we analyzed brain pathology and brain edema. As shown in Figure 1E, compared with the sham group on day 7, rats from TBI and TBI+ vehicle groups showed serious pathological changes. Treatment with NaHS significantly improved the pathological changes. In addition, TBI led to a significant increase in the percentage of brain water content compared to the sham group (Figure 1F). Treatment with NaHS markedly reduced the percentage of brain water content.

### H_2_S inhibited apoptosis after TBI

**Figure 2:**
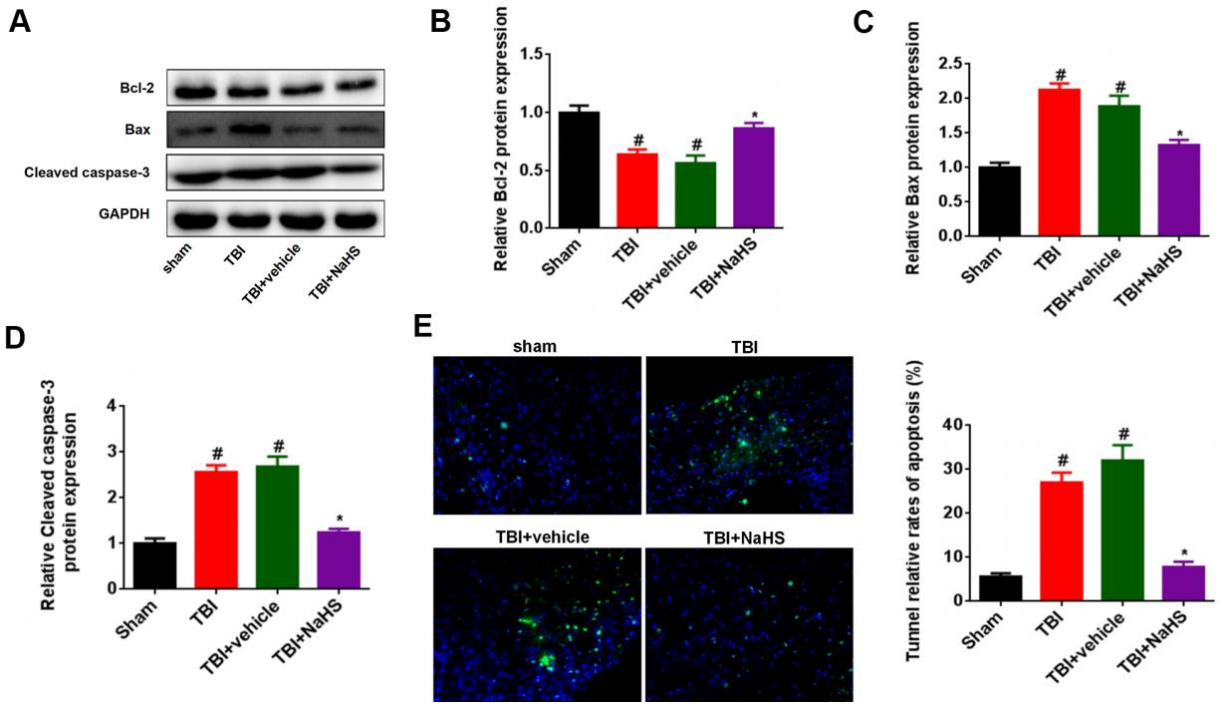
**Treatment with H_2_S inhibited TBI-induced apoptosis.** (**A**) Western blot was performed to analyze the protein level of Bcl-2, Bax, and Cleaved caspase-3 in TBI-challenged rats after treatment with NaHS. (**B**–**D**) The band density of Bcl-2 (**B**), Bax (**C**), and Cleaved caspase-3 (**D**) was analyzed using Image J. (**E**) The apoptosis rate of cerebral cortex in TBI rats was analyzed by Tunnel staining. * P<0.05, vs. TBI and TBI+ vehicle groups. #P<0.05 vs. sham group.

To investigate the effect of H_2_S on apoptosis after TBI, western blot was performed to detect the expression of the apoptosis-associated protein, such as Bcl-2, Bax, and Cleaved caspase-3. As shown in Figure 2A, 2B, the Bcl-2 expression was significantly down-regulated after TBI compared to the sham group. Treatment with NaHS markedly increased the expression of Bcl-2 after TBI. By contrast, treatment with NaHS inhibited the expression of Bax and Cleaved caspase-3, which was increased in TBI-challenged rats (Figure 2A, 2C, 2D). Moreover, Tunnel staining also showed that treatment with NaHS reduced the apoptosis rate of cerebral cortex in TBI rats (Figure 2E).

### H_2_S inhibited the levels of glutamate after TBI

**Figure 3:**
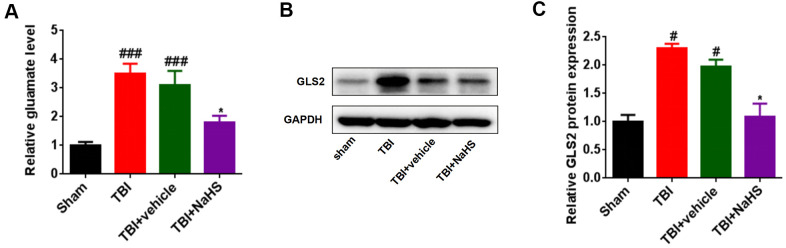
**Treatment with H2S inhibited TBI-induced glutamate.** (**A**) The level of glutamate in TBI-challenged rats after treatment with NaHS. (**B**) Western blot was performed to analyze the protein level of GLS-2 in TBI-challenged rats after treatment with NaHS. (**C**) The band density of GLS-2 was analyzed using Image J. * P<0.05, vs. TBI and TBI+ vehicle groups. #P<0.05, ###P<0.001 vs. sham group.

We then investigated the effect of H_2_S on glutamate levels after TBI. As shown in Figure 3A, the glutamate level was significantly increased after TBI. Treatment with NaHS markedly reduced the glutamate level in TBI-challenged rats. Given that the GLS2 level was regulated by glutaminase, we also determined the effect of H_2_S on GLS2 expression after TBI. The protein expression of GLS2 in TBI-challenged rats was higher than that in the sham group rats. Moreover, the GLS2 protein level was significantly decreased in the TBI+ NaHS group compared to TBI and TBI+ vehicle groups (Figure 3B, 3C).

### H_2_S inhibited brain oxidative stress after TBI

**Figure 4:**
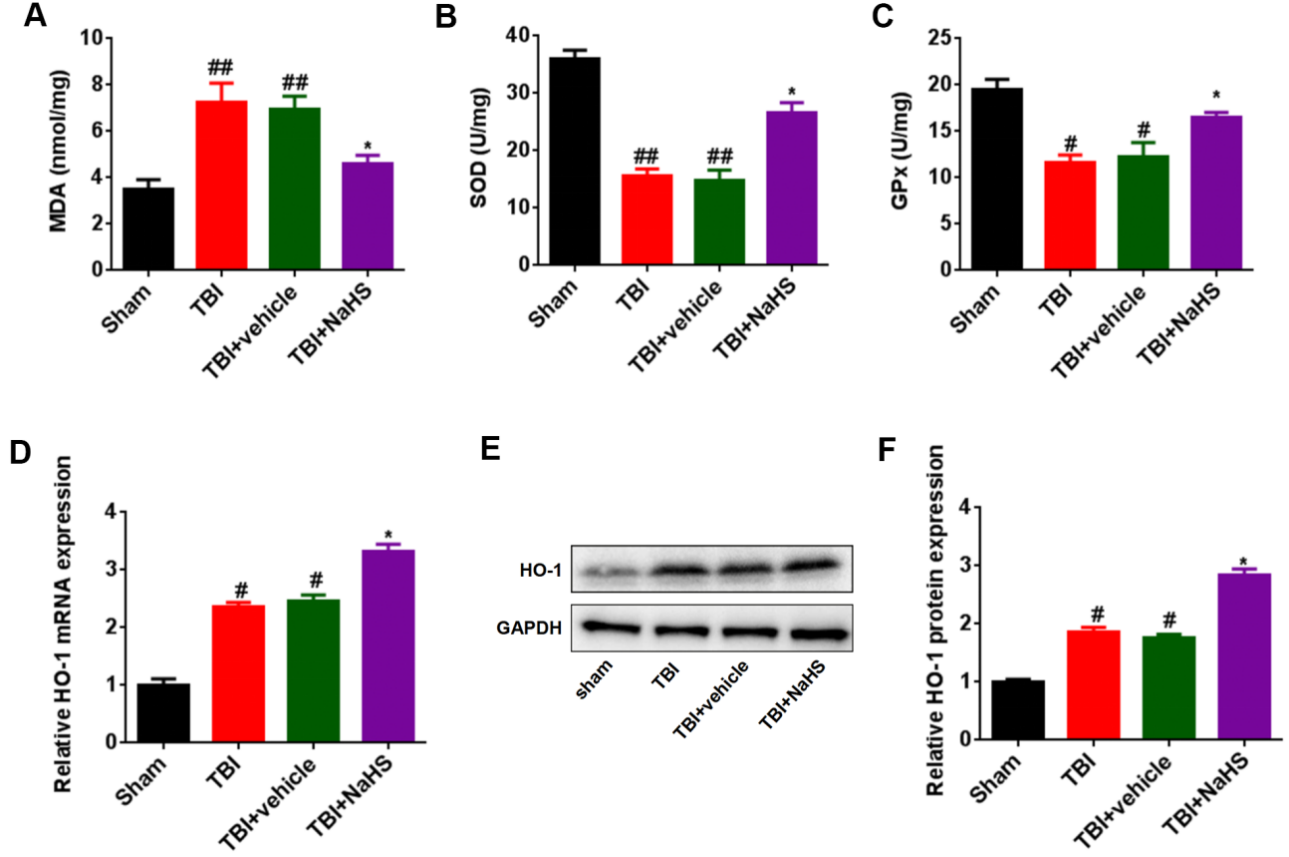
**Treatment with H2S inhibited TBI-induced oxidative stress and HO-1 expression.** (**A**) The level of MDA in TBI-challenged rats after treatment with NaHS. (**B**, **C**) The activities of SOD (**B**) and GPx (**C**) in TBI-challenged rats after treatment with NaHS. (**D**) The mRNA level of HO-1 in TBI-challenged rats after treatment with NaHS. (**E**) The protein level of HO-1 in TBI-challenged rats after treatment with NaHS. (**F**) The band density of HO-1 was analyzed using Image J. * P<0.05, vs. TBI and TBI+ vehicle groups. #P<0.05, ##P<0.01 vs. sham group.

To explore the effect of H_2_S on brain oxidative stress after TBI, malondialdehyde (MDA) content, superoxide dismutases (SOD) activities, and glutathione peroxidase (GPx) activities were assessed, respectively. Compared to the sham group, TBI lead to an increase in the level of MDA and a decrease in the activities of SOD and GPx (Figure 4A–4C). Treatment with NaHS reversed the effect of TBI on MDA content, SOD activities, and GPx activities. Besides, we further analyzed the expression of HO-1, an oxidative stress gene, using RT-qPCR and western blot analysis. The mRNA and protein expression of HO-1 in TBI-challenged rats was higher than that in sham group rats (Figure 4D–4F). Treatment with NaHS increased the mRNA and protein expression of HO-1 in TBI-challenged rats.

### H2S inhibited glutamate-mediated oxidative stress via the p53/GLS-2 pathway

**Figure 5:**
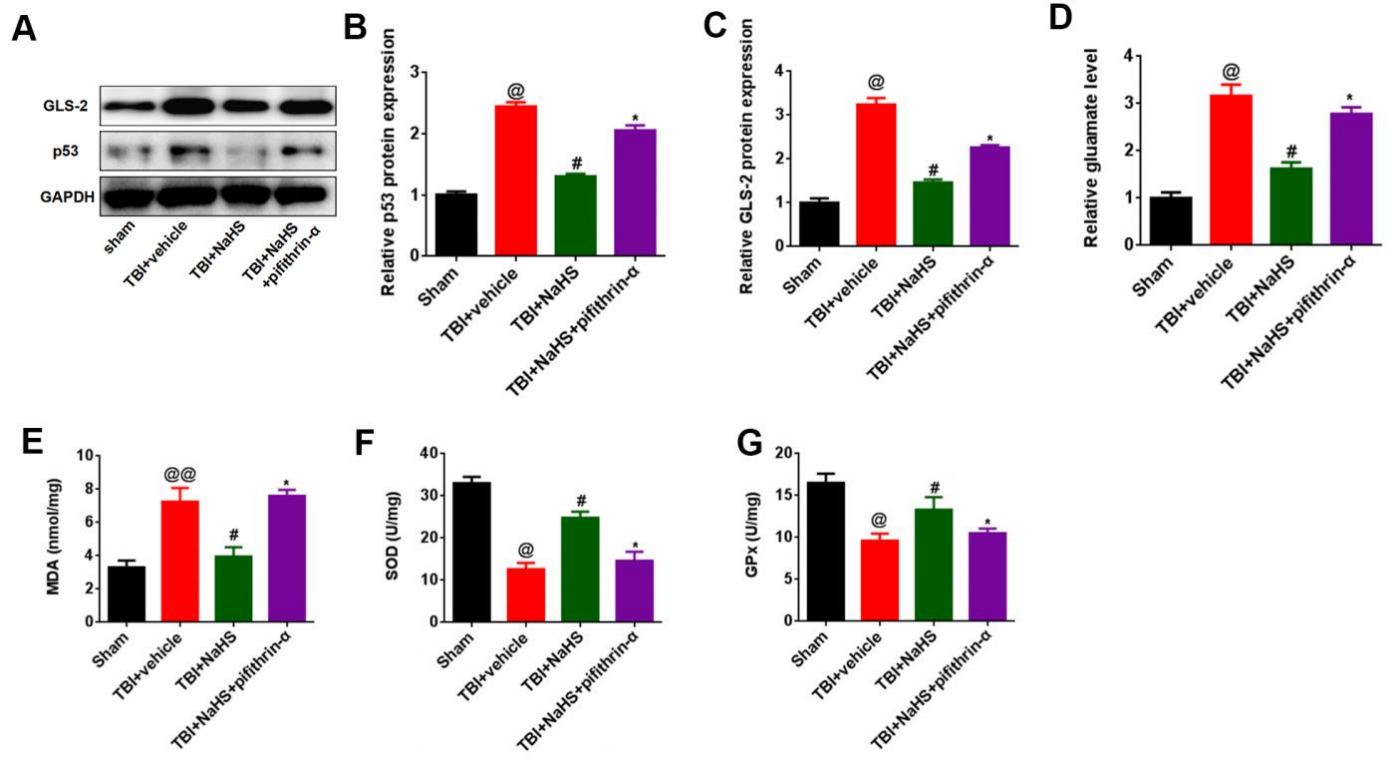
**p53 inhibition reversed the effect of H2S on glutamate and glutamate-mediated oxidative stress after TBI.** (**A**) The protein level of GLS-2 and p53 in TBI-challenged rats after co-treatment with NaHS and pifithrin-α. (**B**, **C**) The band density of GLS-2 (**B**) and p53 (**C**) was analyzed using Image J. (**D**) The level of glutamate in TBI-challenged rats after co-treatment with NaHS and pifithrin-α. (**E**) The level of MDA in TBI-challenged rats after co-treatment with NaHS and pifithrin-α. (**F**, **G**) The activities of SOD (**F**) and GPx (**G**) in TBI-challenged rats after co-treatment with NaHS and pifithrin-α. * P<0.05, vs. TBI+NaHS+ pifithrin-α group. #P<0.05, vs. TBI+ vehicle group. @P<0.05, @@<0.01, vs. sham group.

Since H_2_S can inhibit glutamate and oxidative stress, we hypothesized that the inhibited glutamate and oxidative stress effect was induced by the p53/GLS-2 signaling pathways. As shown in Figure 5A, 5B, treatment with NaHS inhibited the protein expression of p53 in TBI-challenged rats, while pifithrin-α treatment abolished the inhibitory effect of H_2_S on p53 expression in TBI-challenged rats. Furthermore, pifithrin-α treatment reversed the effect of H_2_S on GLS2 and glutamate expression in rats after TBI (Figure 5A, 5C, 5D). More importantly, pifithrin-α treatment abolished the effect of H_2_S on MDA content, SOD activities, and GPx activities in TBI-challenged rats (Figure 5E–5G).

## DISCUSSION

Previous studies have shown the neuroprotective roles of H_2_S on TBI [13, 15]. Mingyang et al. reported that H_2_S pretreatment had reduced brain edema, improved motor performance, and ameliorated performance in the Morris water maze test after TBI [17]. Karimi et al. showed that NaHS has a neuroprotective effect on TBI-induced memory impairment in rats [14]. Also, NaHS treatment ameliorated brain injuries, characterized by an increase of blood-brain barrier permeability, brain edema, and lesion volume, as well as neurologic dysfunction in TBI-challenged rats [16]. In this study, our data also demonstrated that H_2_S alleviated TBI-induced motor and spatial memory deficits, brain pathology, and brain edema. Moreover, we also found that NaHS treatment markedly increased the expression of Bcl-2, while inhibited the expression of Bax and Cleaved caspase-3 in TBI-challenged rats. Tunnel staining also demonstrated this results. These results were similar to the results of previous studies [17, 18], and suggested that H2S played the protective effect against TBI by regulating apoptosis.

It has been well known that glutamate-induced excitotoxicity, which leads to neuronal damage and functional impairments, is implicated in TBI [19]. The metal chaperone, PBT2, currently in clinical trials for Huntington’s disease, could protect against glutamate-induced excitotoxicity thought to underlie both acute and chronic neurodegenerative diseases [20]. Moreover, zolpidem exerted the neuroprotective effect in differentiated P19 neurons by inhibiting the glutamate-induced toxicity [10]. Herein, we hypothesized that H_2_S-mediated neuroprotective effects on TBI may be associated with glutamate-induced toxicity. We found that treatment with NaHS markedly reduced the glutamate and glutaminase protein levels in TBI-challenged rats. These results showed that H_2_S played neuroprotective effects on TBI through inhibiting glutamate-induced toxicity. Jiang et al. found that exogenous H_2_S administered could exert a protective effect against TBI via activation of mitoK(ATP) channels and reduction of oxidative stress [16]. In addition, the protective effect of H_2_S against TBI was associated with regulating apoptosis and autophagy [17]. Therefore, we inferred that H_2_S played neuroprotective effects on TBI via multiple mechanisms such as inhibiting glutamate-induced toxicity, activating of mitoK(ATP) channels, and regulating apoptosis and autophagy.

Oxidative stress and their byproducts could cause brain damage and overall clinical outcome [21]. Nrf2 is an important protective factor against TBI-induced injuries by inhibiting oxidative stress after TBI [22]. PDIA3 provided significant improvements in cognitive impairments and contusion volume induced by TBI through attenuating oxidative stress [23]. Given that glutamate-mediated oxidative stress was associated with multiplication, differentiation, inflammation, survival, and apoptosis of cells [24], we also explored the effect of H_2_S on brain oxidative stress after TBI. We found that treatment with NaHS reversed the effect of TBI on MDA content, SOD activities, and GPx activities, three biomarkers of oxidative stress. Moreover, NaHS treatment inhibited the mRNA and protein expression of HO-1 in TBI-challenged rats. These results suggested that the neuroprotective effects of H_2_S on TBI may be associated with glutamate-mediated oxidative stress.

In addition, we investigated the molecular mechanisms that H_2_S regulated the glutamate-mediated oxidative stress. A previous study showed that the p53 pathway was involved in the progression of TBI. Hong et al. reported that JNK-mediated p53 expression could regulate neuron autophagy following TBI in rats [25]. The p53 inactivator pifithrin-α oxygen analogue could inhibit glutamate-induced excitotoxicity and improve histological and functional outcomes after experimental TBI [26]. Since GLS-2 was a p53 target gene [27], we hypothesized that the inhibited glutamate and oxidative stress effect was induced by the p53/GLS-2 signaling pathways. Our results showed that pifithrin-α treatment reversed the inhibitory effect of H_2_S on p53, GLS-2, and glutamate expression in TBI-challenged rats. Moreover, pifithrin-α treatment abolished the effect of H_2_S on MDA content, SOD activities, and GPx activities in TBI-challenged rats. These results suggested that H_2_S inhibited glutamate-mediated oxidative stress via the p53/GLS-2 pathway.

In conclusion, the present study showed that H_2_S treatment alleviated TBI-induced motor and spatial memory deficits and brain edema, as well as inhibited apoptosis, glutamate-mediated oxidative stress through the p53/GLS-2 pathway.

## MATERIALS AND METHODS

### Mouse TBI model

Sprague-Dawley (SD) rats, weighing 250-300 g, were obtained from the Animal Center of the Hebei Medical University (Shijiazhuang, China). All mouse experiments were approved by the Ethics Committee of The Second Hospital of Hebei Medical University. The mouse TBI model was established as previously described [28]. Briefly, the rats were anesthetized with 4% chloral hydrate. The following surgery was performed under aseptic conditions and mounted in a stereotaxic system (David Kopf Instruments, Tujunga, California). A midline incision was made to expose the skull, and the bone flap was carefully removed by a manual trephine (Roboz Surgical Instrument Co., Gaithersburg, MD). We then used a weight-drop device to perform the TBI in the left part of the brain as described previously [29, 30]. For the sham operation group, rats underwent the same procedure as TBI rats except for the impact.

To examine the effect of H_2_S on TBI, all rats were randomly allocated into sham, TBI, TBI+vehicle, and TBI+NaHS groups. The rats in the TBI+NaHS group were i.p. injected with NaHS (1mmol/kg, Sigma-Aldrich, St. Louis, MO), and the rats in the TBI+ vehicle group were i.p. injected with saline 30 min before TBI.

### Wire-grip test

We used a wire-grip test to evaluate the motor function as described previously [28, 29]. Briefly, we placed rats on the metal wire, which was suspended 45 cm above a foam pad. We then measured and recorded the latency that a mouse remained on the wire within a 60 s’ interval. The scores of wire-grip were calculated as described previously [31].

### Rota-rod test

We also used a rota-rod test to evaluate the motor function as described previously [32]. Before surgery, all rats were pre-trained for balancing on an automated rota-rod (Ugo Basile, Comerio, Italy) at a constant speed of 40 rpm. The average latency to fall from the rod was recorded, and the maximum cutoff time was 180 s.

### Morris water maze test

We performed a Morris water maze test to evaluate spatial learning and memory performance as described previously [28, 33]. Briefly, the water was added into an experimental apparatus, and colored by white non-toxic food pigment. A clear plexiglass goal platform was placed 0.5 cm under the water surface. We allowed rats to find the submerged platform at a maximum of 90 s on days 8 to 15, and remain on the platform for an additional 10 s if the mouse reached the submerged platform. Then, we used a video/compute system to recorded and analyzed the escape latency that a mouse reached the visible platform. For the spatial probe test, we removed the submerged platform and allowed rats to explore the pool within 90 s on day 16. A video tracking system was used to monitor the frequency of passing through the target quadrant.

### Pathological analysis of the brain tissues

For H&E staining, the 5 μm slides from brains of rats in sham, TBI, TBI+vehicle, or TBI+NaHS group were stained by H&E staining kit (Solarbio, China) according to the manufacturer’s instructions. Briefly, the slides were deparaffinized using xylene and ethanol. After permeabilized, the slides were stained with hematoxylin and eosin. Finally, imajes were obtained from an optical microscope.

### Brain water content

A wet-dry weight method was used to measure the brain water content as described previously [28, 33]. Briefly, the whole brains were removed from anesthetized rats and immediately weighed to determine wet weight. The brain tissues were completely dried in an oven at 100° C. After 24h, the dry weight was measured. Brain water content was calculated as (wet weight−dry weight)/wet weight×100%.

### Malondialdehyde (MDA), superoxide dismutases (SOD), and glutathione peroxidase (GPx) assay

The activities of MDA, SOD, and GPx in brain tissue were analyzed using using a commercial kits (Jiangcheng, Nangjing, China, #A003-1-2 for MDA, #A001-4-1 for SOD; #A005-1-2 for GPx) according to the manufacturer’s instructions.

### Western blot

The total protein from cortical was lysed in RIPA lysis with PMSF and protease inhibitor (Thermo Fisher Scientific, USA). The protein concentration was detected using a BCA assay kit (Beyotime, Shanghai China). Equal proteins (30 μg) were resolved by SDS-PAGE and transferred to a polyvinylidene difluoride membrane (Millipore, MA, USA). The membranes were blocked with 5% BSA and were incubated with primary antibodies to anti-Bcl-2 (Abcam), anti-Bax (Abcam), anti-Cleaved caspase-3 (CST), anti-GLS2 (Abcam), anti-HO-1 (CST), anti-p-AKT (CST), anti-t-AKT (CST) and anti-GAPDH (Abcam) overnight at 4° C. Then the membranes were incubated with secondary antibody and visualized using an enhanced chemiluminescence system (Pierce Biotech, IL, USA). The densitometry of each band was quantified by ImageJ software.

### Tunnel apoptosis staining

For Tunnel apoptosis staining, the 5 μm slides from brains of rats in sham, TBI, TBI+vehicle, or TBI+NaHS group were stained by the Tunnel staining kit (Roche, USA) according to the manufacturer’s instructions. Briefly, the slides from brains of rats were dewaxed and rehydrated. Then, the slides were incubated with TUNEL reaction mixture at 37° C for 1 h. The nuclei were stained with DAPI.

### RNA isolation and quantitative real-time PCR (RT-qPCR) assays

Total RNA was extracted from cortical using TRIzol reagent (Invitrogen, Carlsbad, CA, USA) according to the manufacturer’s instructions. The method used to detect HO-1 mRNA expression was based on a previous report [22]. Briefly, cDNA was constructed using a commercial kit (GeneCopoeia, Rockville, USA). Then, RT-PCR was performed on an Applied Biosystems 7500 Sequence Detection system using a SYBR PrimeScript RT-qPCR Kit (Takara). The primers used in this study were as follows: HO-1 Forward Primer 5’- CTGTGCCACCTGGAACTGAC -3’, Reverse Primer 5’- TCTTGTGGGTCTTGAGCTGTT -3’; β-Actin Forward Primer 5’-GTTGAGAACCGTGTACCATGT-3’, Reverse Primer 5’-TTCCCACAATTTGGCAAGAGC-3’. β-Actin was used as an internal control.

### Statistical analysis

All data were present as mean±SD. Student t-test was used to determine statistically significant differences between two groups, and one-way ANOVA was used for > 2 groups. P<0.05 was considered to be statistically significant.

## References

[1] Jiang JY, Gao GY, Feng JF, Mao Q, Chen LG, Yang XF, Liu JF, Wang YH, Qiu BH, Huang XJ. Traumatic brain injury in China. Lancet Neurol. 2019; 18:286–95. 10.1016/S1474-4422(18)30469-1 30784557

[2] Mollayeva T, Mollayeva S, Colantonio A. Traumatic brain injury: sex, gender and intersecting vulnerabilities. Nat Rev Neurol. 2018; 14:711–22. 10.1038/s41582-018-0091-y 30397256

[3] Murray NM, Threlkeld ZD, Hirsch KG. Will we ever make headway in severe traumatic brain injury treatment trials? JAMA Neurol. 2020; 77:411–12. 10.1001/jamaneurol.2019.4672 31961381

[4] Maiden MJ, Cameron PA, Rosenfeld JV, Cooper DJ, McLellan S, Gabbe BJ. Long-term outcomes after severe traumatic brain injury in older adults. A registry-based cohort study. Am J Respir Crit Care Med. 2020; 201:167–77. 10.1164/rccm.201903-0673OC 31657946

[5] Fridman EA, Osborne JR, Mozley PD, Victor JD, Schiff ND. Presynaptic dopamine deficit in minimally conscious state patients following traumatic brain injury. Brain. 2019; 142:1887–93. 10.1093/brain/awz118 31505542 PMC6598636

[6] Dorsett CR, McGuire JL, DePasquale EA, Gardner AE, Floyd CL, McCullumsmith RE. Glutamate neurotransmission in rodent models of traumatic brain injury. J Neurotrauma. 2017; 34:263–72. 10.1089/neu.2015.4373 27256113 PMC5220558

[7] Guerriero RM, Giza CC, Rotenberg A. Glutamate and GABA imbalance following traumatic brain injury. Curr Neurol Neurosci Rep. 2015; 15:27. 10.1007/s11910-015-0545-1 25796572 PMC4640931

[8] Garzón F, Coimbra D, Parcerisas A, Rodriguez Y, García JC, Soriano E, Rama R. NeuroEPO preserves neurons from glutamate-induced excitotoxicity. J Alzheimers Dis. 2018; 65:1469–83. 10.3233/JAD-180668 30175978

[9] MacMullin P, Hodgson N, Damar U, Lee HH, Hameed MQ, Dhamne SC, Hyde D, Conley GM, Morriss N, Qiu J, Mannix R, Hensch TK, Rotenberg A. Increase in seizure susceptibility after repetitive concussion results from oxidative stress, parvalbumin-positive interneuron dysfunction and biphasic increases in glutamate/GABA ratio. Cereb Cortex. 2020; 30:6108–20. 10.1093/cercor/bhaa157 32676666 PMC8248830

[10] Jazvinšćak Jembrek M, Radovanović V, Vlainić J, Vuković L, Hanžić N. Neuroprotective effect of zolpidem against glutamate-induced toxicity is mediated via the PI3K/Akt pathway and inhibited by PK11195. Toxicology. 2018; 406:58–69. 10.1016/j.tox.2018.05.014 29859204

[11] Chan SJ, Wong PT. Hydrogen sulfide in stroke: Protective or deleterious? Neurochem Int. 2017; 105:1–10. 10.1016/j.neuint.2016.11.015 28174023

[12] Kamat PK, Kyles P, Kalani A, Tyagi N. Hydrogen sulfide ameliorates homocysteine-induced Alzheimer’s disease-like pathology, blood-brain barrier disruption, and synaptic disorder. Mol Neurobiol. 2016; 53:2451–67. 10.1007/s12035-015-9212-4 26019015 PMC4662933

[13] Kumar M, Sandhir R. Hydrogen sulfide in physiological and pathological mechanisms in brain. CNS Neurol Disord Drug Targets. 2018; 17:654–70. 10.2174/1871527317666180605072018 29866024

[14] Karimi SA, Hosseinmardi N, Janahmadi M, Sayyah M, Hajisoltani R. The protective effect of hydrogen sulfide (H_2_S) on traumatic brain injury (TBI) induced memory deficits in rats. Brain Res Bull. 2017; 134:177–82. 10.1016/j.brainresbull.2017.07.014 28739248

[15] Zhang J, Shi C, Wang H, Gao C, Chang P, Chen X, Shan H, Zhang M, Tao L. Hydrogen sulfide protects against cell damage through modulation of PI3K/Akt/Nrf2 signaling. Int J Biochem Cell Biol. 2019; 117:105636. 10.1016/j.biocel.2019.105636 31654751

[16] Jiang X, Huang Y, Lin W, Gao D, Fei Z. Protective effects of hydrogen sulfide in a rat model of traumatic brain injury via activation of mitochondrial adenosine triphosphate-sensitive potassium channels and reduction of oxidative stress. J Surg Res. 2013; 184:e27–35. 10.1016/j.jss.2013.03.067 23590867

[17] Zhang M, Shan H, Chang P, Wang T, Dong W, Chen X, Tao L. Hydrogen sulfide offers neuroprotection on traumatic brain injury in parallel with reduced apoptosis and autophagy in mice. PLoS One. 2014; 9:e87241. 10.1371/journal.pone.0087241 24466346 PMC3900713

[18] Xu K, Wu F, Xu K, Li Z, Wei X, Lu Q, Jiang T, Wu F, Xu X, Xiao J, Chen D, Zhang H. NaHS restores mitochondrial function and inhibits autophagy by activating the PI3K/Akt/mTOR signalling pathway to improve functional recovery after traumatic brain injury. Chem Biol Interact. 2018; 286:96–105. 10.1016/j.cbi.2018.02.028 29567101

[19] Vincenzi F, Pasquini S, Gessi S, Merighi S, Romagnoli R, Borea PA, Varani K. The detrimental action of adenosine on glutamate-induced cytotoxicity in PC12 cells can be shifted towards a neuroprotective role through A_1_ AR positive allosteric modulation. Cells. 2020; 9:1242. 10.3390/cells9051242 32443448 PMC7290574

[20] Johanssen T, Suphantarida N, Donnelly PS, Liu XM, Petrou S, Hill AF, Barnham KJ. PBT2 inhibits glutamate-induced excitotoxicity in neurons through metal-mediated preconditioning. Neurobiol Dis. 2015; 81:176–85. 10.1016/j.nbd.2015.02.008 25697105

[21] Marklund N, Clausen F, Lewander T, Hillered L. Monitoring of reactive oxygen species production after traumatic brain injury in rats with microdialysis and the 4-hydroxybenzoic acid trapping method. J Neurotrauma. 2001; 18:1217–27. 10.1089/089771501317095250 11721740

[22] Zhang M, Huang LL, Teng CH, Wu FF, Ge LY, Shi YJ, He ZL, Liu L, Jiang CJ, Hou RN, Xiao J, Zhang HY, Chen DQ. Isoliquiritigenin Provides Protection and Attenuates Oxidative Stress-Induced Injuries via the Nrf2-ARE Signaling Pathway After Traumatic Brain Injury. Neurochem Res. 2018; 43:2435–45. 10.1007/s11064-018-2671-z 30446968

[23] Wang WT, Sun L, Sun CH. PDIA3-regulted inflammation and oxidative stress contribute to the traumatic brain injury (TBI) in mice. Biochem Biophys Res Commun. 2019; 518:657–63. 10.1016/j.bbrc.2019.08.100 31466719

[24] Kim JJ, Kang YJ, Shin SA, Bak DH, Lee JW, Lee KB, Yoo YC, Kim DK, Lee BH, Kim DW, Lee J, Jo EK, Yuk JM. Phlorofucofuroeckol improves glutamate-induced neurotoxicity through modulation of oxidative stress-mediated mitochondrial dysfunction in PC12 cells. PLoS One. 2016; 11:e0163433. 10.1371/journal.pone.0163433 27669570 PMC5036853

[25] Hong MY, Gao JL, Cui JZ, Wang KJ, Tian YX, Li R, Wang HT, Wang H. Effect of c-Jun NH_2_-terminal kinase-mediated p53 expression on neuron autophagy following traumatic brain injury in rats. Chin Med J (Engl). 2012; 125:2019–24. 22884071

[26] Yang LY, Chu YH, Tweedie D, Yu QS, Pick CG, Hoffer BJ, Greig NH, Wang JY. Post-trauma administration of the pifithrin-α oxygen analog improves histological and functional outcomes after experimental traumatic brain injury. Exp Neurol. 2015; 269:56–66. 10.1016/j.expneurol.2015.03.015 25819102 PMC5193498

[27] Hu W, Zhang C, Wu R, Sun Y, Levine A, Feng Z. Glutaminase 2, a novel p53 target gene regulating energy metabolism and antioxidant function. Proc Natl Acad Sci USA. 2010; 107:7455–60. 10.1073/pnas.1001006107 20378837 PMC2867677

[28] Li Q, Wu X, Yang Y, Zhang Y, He F, Xu X, Zhang Z, Tao L, Luo C. Tachykinin NK1 receptor antagonist L-733,060 and substance P deletion exert neuroprotection through inhibiting oxidative stress and cell death after traumatic brain injury in mice. Int J Biochem Cell Biol. 2019; 107:154–65. 10.1016/j.biocel.2018.12.018 30593954

[29] Luo CL, Chen XP, Yang R, Sun YX, Li QQ, Bao HJ, Cao QQ, Ni H, Qin ZH, Tao LY. Cathepsin B contributes to traumatic brain injury-induced cell death through a mitochondria-mediated apoptotic pathway. J Neurosci Res. 2010; 88:2847–58. 10.1002/jnr.22453 20653046

[30] Luo CL, Li BX, Li QQ, Chen XP, Sun YX, Bao HJ, Dai DK, Shen YW, Xu HF, Ni H, Wan L, Qin ZH, Tao LY, Zhao ZQ. Autophagy is involved in traumatic brain injury-induced cell death and contributes to functional outcome deficits in mice. Neuroscience. 2011; 184:54–63. 10.1016/j.neuroscience.2011.03.021 21463664

[31] Bermpohl D, You Z, Korsmeyer SJ, Moskowitz MA, Whalen MJ. Traumatic brain injury in mice deficient in bid: effects on histopathology and functional outcome. J Cereb Blood Flow Metab. 2006; 26:625–33. 10.1038/sj.jcbfm.9600258 16395279

[32] Wang L, Wang L, Dai Z, Wu P, Shi H, Zhao S. Lack of mitochondrial ferritin aggravated neurological deficits via enhancing oxidative stress in a traumatic brain injury murine model. Biosci Rep. 2017; 37:BSR20170942. 10.1042/BSR20170942 28963372 PMC5672084

[33] Fang J, Wang H, Zhou J, Dai W, Zhu Y, Zhou Y, Wang X, Zhou M. Baicalin provides neuroprotection in traumatic brain injury mice model through Akt/Nrf2 pathway. Drug Des Devel Ther. 2018; 12:2497–508. 10.2147/DDDT.S163951 30127597 PMC6089097

